# A glimpse of antimicrobial resistance gene diversity in kefir and yoghurt

**DOI:** 10.1038/s41598-020-80444-5

**Published:** 2020-12-31

**Authors:** Adrienn Gréta Tóth, István Csabai, Gergely Maróti, Ákos Jerzsele, Attila Dubecz, Árpád V. Patai, Maura Fiona Judge, Sára Ágnes Nagy, László Makrai, Krisztián Bányai, Géza Szita, Norbert Solymosi

**Affiliations:** 1 Centre for Bioinformatics, University of Veterinary Medicine Budapest, Budapest, 1078 Hungary; 2 Department of Phyisics of Complex Systems, Eötvös Loránd University, Budapest, 1117 Hungary; 3 Institute of Plant Biology, Biological Research Center, Szeged, 6726 Hungary; 4 Faculty of Water Sciences, University of Public Service, Baja, 6050 Hungary; 5 Department of Pharmacology and Toxicology, University of Veterinary Medicine Budapest, Budapest, 1078 Hungary; 6 Department of Surgery, Paracelsus Medical University, Nuremberg, 90419 Germany; 7 Department of Internal Medicine and Hematology, Semmelweis University, Budapest, 1088 Hungary; 8 Interdisciplinary Gastroenterology (IGA) Working Group, Semmelweis University, Budapest, 1085 Hungary; 9 Department of Microbiology and Infectious Diseases, University of Veterinary Medicine Budapest, Budapest, 1143 Hungary; 10 Institute for Veterinary Medical Research, Centre for Agricultural Research, Budapest, 1143 Hungary; 11 Department of Food Hygiene, University of Veterinary Medicine Budapest, Budapest, 1078 Hungary

**Keywords:** Nutrition, Risk factors, Antimicrobial resistance, Bacterial genomics, Antimicrobial resistance

## Abstract

Antimicrobial resistance (AMR) is a global threat gaining more and more practical significance every year. The main determinants of AMR are the antimicrobial resistance genes (ARGs). Since bacteria can share genetic components via horizontal gene transfer, even non-pathogenic bacteria may provide ARG to any pathogens which they become physically close to (e.g. in the human gut). In addition, fermented food naturally contains bacteria in high amounts. In this study, we examined the diversity of ARG content in various kefir and yoghurt samples (products, grains, bacterial strains) using a unified metagenomic approach. We found numerous ARGs of commonly used fermenting bacteria. Even with the strictest filter restrictions, we identified ARGs undermining the efficacy of aminocoumarins, aminoglycosides, carbapenems, cephalosporins, cephamycins, diaminopyrimidines, elfamycins, fluoroquinolones, fosfomycins, glycylcyclines, lincosamides, macrolides, monobactams, nitrofurans, nitroimidazoles, penams, penems, peptides, phenicols, rifamycins, tetracyclines and triclosan. In the case of gene lmrD, we detected genetic environment providing mobility of this ARG. Our findings support the theory that during the fermentation process, the ARG content of foods can grow due to bacterial multiplication. The results presented suggest that the starting culture strains of fermented foods should be monitored and selected in order to decrease the intake of ARGs via foods.

## Introduction

Antimicrobial resistance (AMR) is a global threat gaining more and more practical significance every year. Although antimicrobial resistance genes (ARGs) have been present ever since the appearance of the first living microorganisms^1^, the threat arising from AMR has stemmed from the daily use of antibiotics in human and animal healthcare. Even though antibiotic compounds are not directly responsible for the genetic changes behind the appearance of antimicrobial resistance, they place selective pressure towards the amplification of individual bacterial ARGs. Identifying the sources of intake of bacteria carrying ARGs is nowadays a biomedical priority. Bacteria appear in the newborn body right from birth^2^, and later on, their invasion continues from the environment, from other humans and animals, or raw^3^ or processed^4^ food^5^. Bacteria reaching our gut through alimentation may share functional ARGs either with saprophytes or with pathogens in their physical proximity due to horizontal gene transfer (HGT). In the latter case, resistant or multi-resistant pathogenic strains may evolve, and therapeutic options for the treatment of bacterial infections may narrow. By the production and consumption of livestock products, the opportunity to establish transfer connections between bacteria is provided, as bacterial populations with different sets of ARGs may meet. Therefore, popular probiotic products (such as yoghurt and kefir), have the potential to allow encounters between their bacterial strains and those in the consumer. Yoghurt and kefir are probiotic foods with minor differences in their processing steps. Yoghurt is fermented with bacteria, whereas the production of kefir requires fungi in addition. They have both been present in the human diet for a long time and still stand their ground in today’s demanding, health-conscious society. Nevertheless, besides the health benefits, consumption of probiotic food may have an adverse effect. Along with the multiplication of bacteria during the fermentation process, the bacterial resistome also grows. If the intake of probiotic products occurs alongside the right triggers, a higher possibility of HGT is provided in the human gut. Our study examined the diversity of the ARG content of kefir and yoghurt products, their grains and bacterial strains using a unified metagenomic approach.

## Results

The results were organised as follows. After describing the bacteriome of the samples, their resistome and mobilome are shown. This is followed by the summary of abundance changes during fermentation of some ARGs and the related bacteria carrying those ARGs. Finally, we present the non-nudged ARG findings.

### Bacteriome

**Figure 1:**
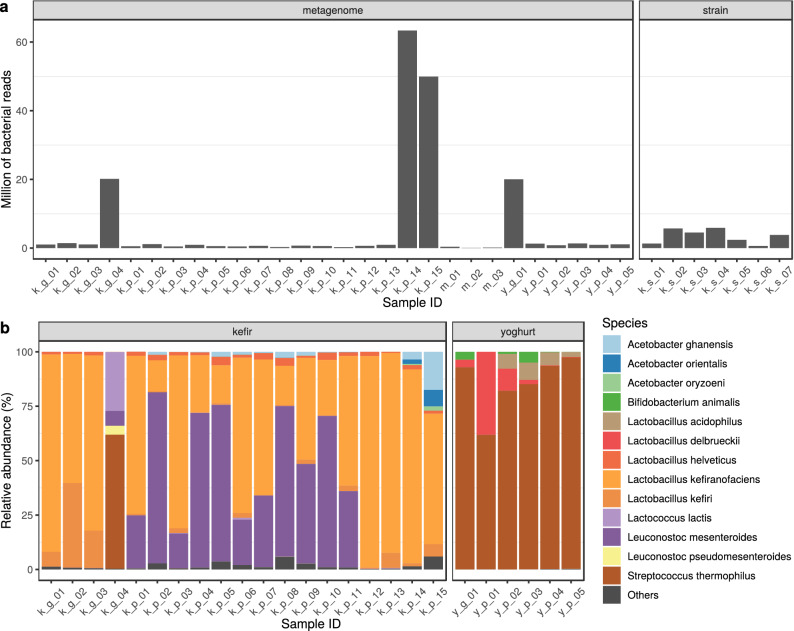
Bacterial content of the samples. (**a**) The number of reads classified bacterial by Kraken2 on the NCBI NT database. Metagenome includes the samples deriving from grains, milk or products. (**b**) Relative abundances of the most common bacterial species in the grain and product samples.

When classified by taxon, the number of reads aligning to bacterial genomes differed in the various samples (Fig. 1a). Two samples (k_g_04 and y_g_01 from bioproject PRJNA644779) contained $\sim$ 20 million reads of bacterial origin. From bioproject PRJNA388572, sample k_p_15 had $\sim$ 50 million bacterial reads, while k_p_14 contained more than 63 million. Excluding these four extremities, the average bacterial read count of the metagenomic samples was $6.7\times 10^5$ (ranging between $7.3\times 10^4$ and $1.4\times 10^6$). The median sequencing depth of the strain k_s_01, k_s_02, k_s_03, k_s_04, k_s_05, k_s_06, k_s_07 were 46, 119, 115, 111, 6, 54, 108, respectively.

Figure 1b. demonstrates the relative abundances of the dominant bacterial species identified in the samples. 99% of all bacteria identified were related to these species. In kefir grains the dominant species were a *Lactobacillus kefiranofaciens* ($57.7\% \pm 40.5\%$), *Lactobacillus kefiri* ($15.7\% \pm 17\%$), *Streptococcus thermophilus* ($15.4\% \pm 30.8\%$), *Lactococcus lactis* ($6.8\% \pm 13.5\%$), *Leuconostoc mesenteroides* ($1.7\% \pm 3.4\%$), *Leuconostoc pseudomesenteroides* ($1\% \pm 2\%$) and *Lactobacillus helveticus* ($1\% \pm 0.7\%$) in descending order of abundance. The most significant species in the products overlapped with those in the kefir grains, although they had differences in their relative abundance (*L. kefiranofaciens* ($55.4\% \pm 29\%$), *L. mesenteroides* ($35.7\% \pm 30\%$), *Acetobacter ghanensis* ($2.1\% \pm 4.4\%$), *L. helveticus* ($2.1\% \pm 1\%$), *L. kefiri* ($1.8\% \pm 2\%$), *Acetobacter orientalis* ($0.6\% \pm 2\%$), *Acetobacter oryzoeni* ($0.2\% \pm 0.5\%$)). The one yoghurt grain examined was dominated by *Streptococcus thermophilus* ($92.8\%$), *Bifidobacterium animalis* ($3.6\%$) and *Lactobacillus delbrueckii* ($3.5\%$) while the core bacteriome of the yoghurt product consisted of *S. thermophilus* ($83.9\% \pm 13.8\%$), *L. delbrueckii* ($10.1\% \pm 16.2\%$), *Lactobacillus acidophilus* ($4.6\% \pm 3.3\%$) and *B. animalis* ($1.2\% \pm 2.1\%$).

### Resistome

**Figure 2:**
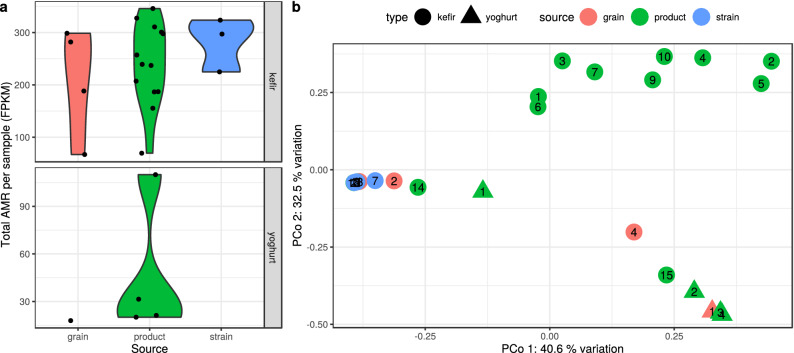
Antimicrobial resistance (AMR) abundance of the samples. (**a**) Violin plot representing the distribution of the total AMR fragments per kilobase per million fragments (FPKM) per sample, grouped by type and source. The horizontally jittered dots represent the FPKM of the samples. (**b**) The AMR abundance diversity ($\beta$-diversity) of the samples. It is plotted on the first two axes of principal coordinate analysis (PCoA) performed on Bray-Curtis distance which was calculated using the relative abundances of contigs harbouring ARGs. The symbols show the type, the colours the source, while the numbers correspond to the sequence number in the Sample ID. Some samples (k_s_01, k_s_04, k_s_05, k_s_06, k_p_08) are not shown as their ORFs did not meet filtering criteria.

**Figure 3:**
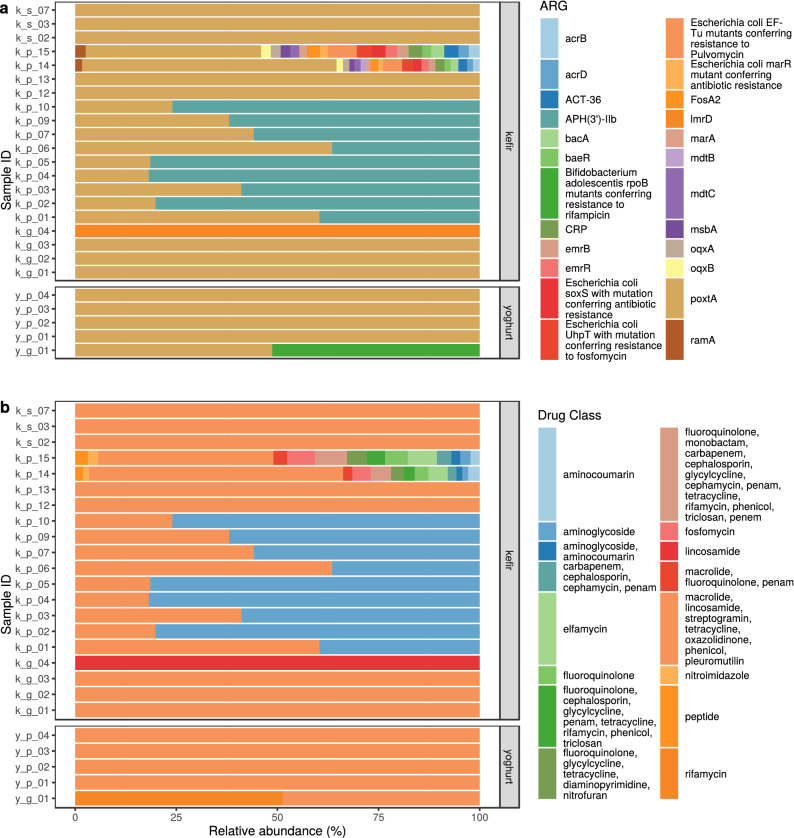
Antimicrobial resistance (AMR) abundance in kefir and yoghurt samples. (**a**) Relative abundance of AMR genes. ORFs having at least 60% length and 90% base sequence identity with the reference ARG sequence are shown. Some samples (k_s_01, k_s_04, k_s_05, k_s_06, k_p_08) are not shown as their ORFs did not meet filtering criteria. (**b**) Relative abundance of drug classes related to the ARGs identified in the samples.

According to our findings based on perfect and strict matches, AMR gene abundances show a great diversity in various types and sources of samples (Fig. 2a). Samples (k_s_01, k_s_04, k_s_05, k_s_06, k_p_08) that did not meet the ORF filtering criteria were not plotted on Figs. 2 and 3. The highest ARG abundance was observed in the kefir strain samples (average: 282 fragments per kilobase per million fragments (FPKM), sd: 51.1) followed by the kefir product ($240 \pm 78.6$) and the kefir grains ($209 \pm 106$). The yoghurt samples had lower abundances, in the only one grain, FPKM was 17.9, while in the products we found $45.7 \pm 32.2$.

A Bray-Curtis distance-based principal coordinate analysis (PCoA) was performed to gain insight into the dissimilarity of the sample ARG abundances (Fig. 2b). With a permutational multivariate analysis of variance on the same distance matrix, we found that the type of the sample explains the 22.17% ($p<0.001$) of dissimilarity among the sample resistomes. For the source grouping, the same measure was 18.92% ($p<0.001$). Based on Fig. 2b one might conclude that the strongest effect on the dissimilarity is the bioproject of origin, as the analysis showed that it explains 35.56% ($p<0.001$) of the dissimilarity variances.

**Figure 4:**
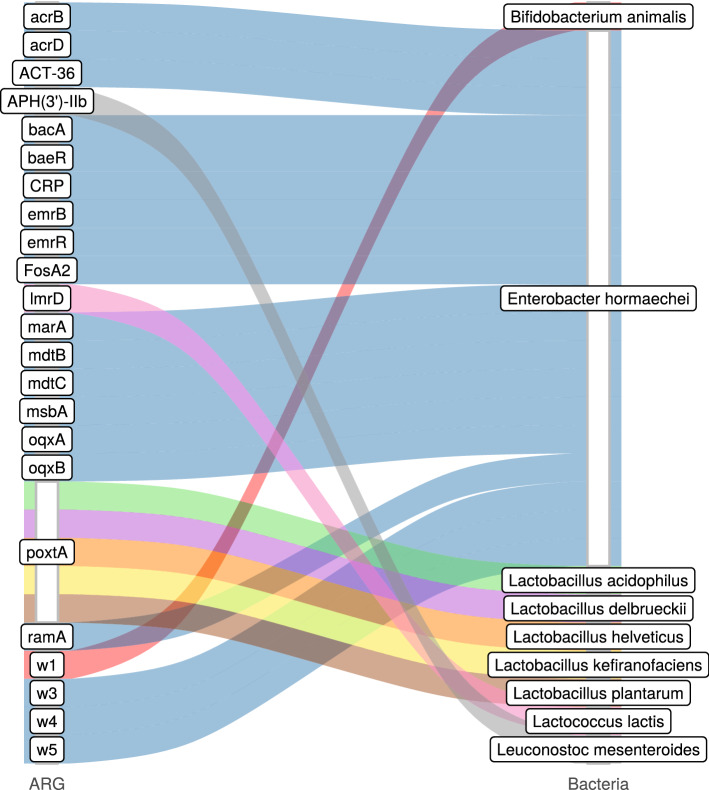
Identified ARGs and their most probable bacteria of origin. The gene names that are too long have been abbreviated (w1: *Bifidobacterium adolescentis* *rpoB* mutants conferring resistance to rifampicin; w3: *Escherichia coli* *marR* mutant conferring antibiotic resistance; w4: *E. coli* *soxS* with mutation conferring antibiotic resistance; w5: *E. coli* *UhpT* with mutation conferring resistance to fosfomycin).

In our kefir samples, we identified 22 ARGs in the product, 2 in the grain and 1 in the strain. In yoghurt, there was 1 ARG in the product and 2 in the grain (Fig. 3a). The relative abundances of antibiotic classes affected are shown in Fig. 3b for each sample. The detected ARGs and their most probable bacteria of origin are summarized on Fig. 4. The kefir ARGs identified in the product may help bacteria in the defence against aminocoumarins, aminoglycosides, carbapenems, cephalosporins, cephamycins, diaminopyrimidines, elfamycins, fluoroquinolones, fosfomycins, glycylcyclines, lincosamides, macrolides, monobactams, nitrofurans, nitroimidazoles, oxazolidinones, penams, penems, peptides, phenicols, pleuromutilins, rifamycins, streptogramins, tetracyclines and triclosan. Contigs containing these ARGs belonged to the genomes of *Enterobacter hormaechei* (genes: *acrB*; *acrD*; *ACT-36*; *bacA*; *baeR*; *CRP*; *emrB*; *emrR*; *Escherichia coli* *marR* mutant conferring antibiotic resistance; *E. coli* *soxS* with mutation conferring antibiotic resistance; *E. coli* *UhpT* with mutation conferring resistance to fosfomycin; *FosA2*; *marA*; *mdtB*; *mdtC*; *msbA*; *oqxA*; *oqxB*; *ramA*), *L. helveticus* (gene *poxtA*), *L. kefiranofaciens* (gene *poxtA*) and *L. mesenteroides* (gene *APH(3’)-IIb*). ARGs originating from the kefir grain may play a role in the appearance of resistance against lincosamides, macrolides, oxazolidinones, phenicols, pleuromutilins, streptogramins and tetracyclines and were found in the genomes of *L. kefiranofaciens* (gene *poxtA*) and *L. lactis* (gene *lmrD*). Gene *poxtA* deriving from kefir strains (*L. kefiranofaciens* and *L. plantarum*) confers resistance against lincosamides, macrolides, oxazolidinones, phenicols, pleuromutilins, streptogramins and tetracyclines. Genes found in yoghurt grains encoded resistance against lincosamides, macrolides, oxazolidinones, phenicols, pleuromutilins, rifamycins, streptogramins and tetracyclines, while the ARGs of the product itself may weaken the efficacy of lincosamides, macrolides, oxazolidinones, phenicols, pleuromutilins, streptogramins and tetracyclines. Contigs involving ARGs of the yoghurt product could have been connected to *L. acidophilus* (gene *poxtA*) and *L. delbrueckii* (gene *poxtA*). However, the ARGs of the grains aligned to the reference sequence of *B. animalis* (gene *Bifidobacterium adolescentis* *rpoB* mutants conferring resistance to rifampicin) and *L. delbrueckii* (gene *poxtA*).

Based on the ARG abundances the proportion of resistance mechanisms was calculated for each sample. In the kefir product samples the dominant mechanism of identified ARGs was the antibiotic target protection (50.73%), followed by antibiotic inactivation (45.45%), antibiotic efflux (2.03%), antibiotic target alteration (1.07%), antibiotic efflux; reduced permeability to antibiotic (0.32%), antibiotic target alteration; antibiotic efflux; reduced permeability to antibiotic (0.27%), antibiotic target alteration and antibiotic efflux (0.13%). In the kefir grain samples, the main mechanisms were antibiotic target protection (91.98%) and antibiotic efflux (8.02%). In the kefir strains and yoghurt products, the antibiotic target protection was the only mechanism detected. In the one yoghurt grain sample, antibiotic target alteration; antibiotic target replacement (51.28%) and antibiotic target protection (48.72%) are the possible resistance mechanisms.

### Mobile elements

The results of mobile genetic element (MGE) domain coexisting analysis showed that the ARG *lmrD* in sample k_g_04 might be mobile since the contig containing the gene had a transposase ORF within the distance of 10 ORFs. According to the analysis executed with PlasFlow^6^ there were not any identifiable contigs with plasmid origins harbouring ARGs.

### ARG abundance changes during kefir fermentation

**Figure 5:**
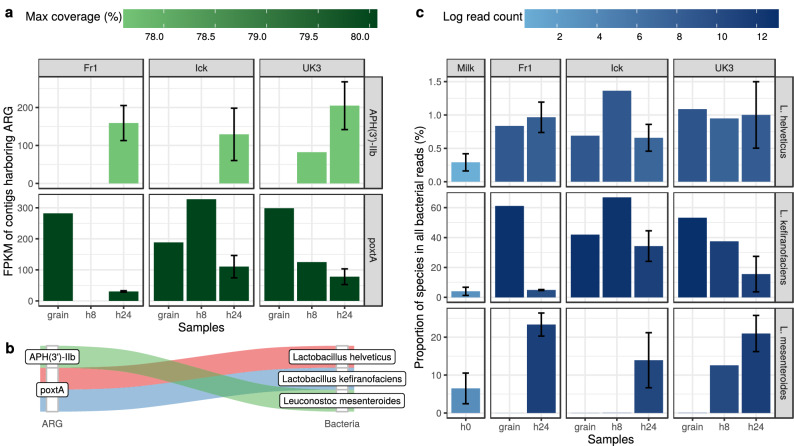
Changes during kefir fermentation. (**a**) Antimicrobial resistance gene (ARG) abundance expressed as fragments per kilobase per million fragments (FPKM) based on the alignment of bacterial reads to the ARG harbouring contigs. (**b**) ARGs and their most likely origins. (**c**) Relative abundances of bacteria with a probable ARG content.

According to the metagenomic analysis published by Walsh et al.^7^, ARG abundances change during the fermentation process (Fig. 5a). In the case of all three grains (Fr1, Ick, UK3) *APH(3’)-IIb* is present in the kefirs fermented for 24 hours, while it is absent in all other time phases except for sample UK3 after 8 hours. *PoxtA* was detectable in all samples except the 8 hour Fr1 kefir sample. The abundance fold change of 24 hours with respect to grain samples was 0.10, 0.59 and 0.26 for the starter culture Fr1, Ick and Uk3, respectively. Between the hour 8 and 24 samples, *poxtA* abundance showed a 0.34-fold change in the Ick kefir, while in the case of the UK3 kefir sequence this value reached 0.62.

Contigs harbouring ARGs were classified taxonomically (Fig. 5c). All contigs containing the gene *APH(3’)-IIb* were assigned to *L. mesenteroides*. Contigs with *poxtA* were assigned to the reference genome of *L. helveticus* and *L. kefiranofaciens*.

All bacterial reads were then aligned to the reference genomes of bacteria mentioned above, and the hits are expressed proportionally (Fig. 5b). In contrast to *L. kefiranofaciens* that showed a decreasing tendency, an increase in time is observable by the relative abundances of *L. mesenteroides*. The proportion of reads assigned to *L. helveticus* shows no tendential increase or decrease in time. The increase of abundance of *APH(3’)-IIb* shows a positive association with the relative abundance of *L. mesenteroides*. Similarly, *poxtA* abundance is decreasing with the relative abundance of *L. kefiranofaciens*.

### Excluding nudged hits

**Figure 6:**
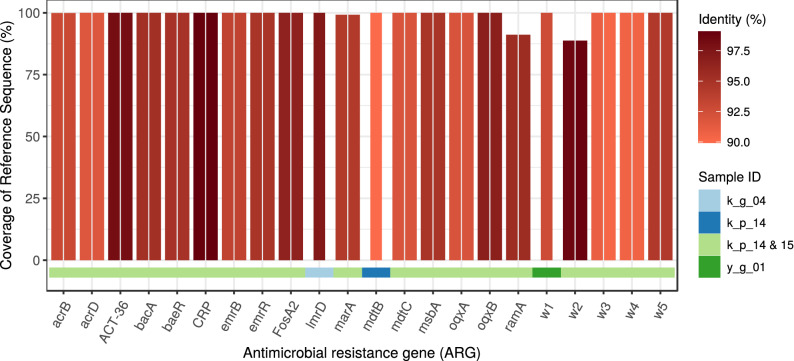
Identified ARGs excluding nudged findings. The coverage and identity of detected open reading frames (ORFs) by antimicrobial resistance genes (ARGs). The ORF covered proportion of the reference ARG sequence and the identity % of predicted protein (colour). The gene names which are too long have been abbreviated (w1: *Bifidobacterium adolescentis* *rpoB* mutants conferring resistance to rifampicin; w2: *Escherichia coli* *EF-Tu* mutants conferring resistance to Pulvomycin; w3: *E. coli* *marR* mutant conferring antibiotic resistance; w4: *E. coli* *soxS* with mutation conferring antibiotic resistance; w5: *E. coli* *UhpT* with mutation conferring resistance to fosfomycin).

In order to set the alignment restrictions of ORFs to reference ARGs even stricter, we selected for a subgroup of reference ARGs that fit the ORFs from the starting base position on. Thus, nudging on the reference sequence by the alignment was avoided. With such a shrinkage, we reduced the number of detectable ARGs to four samples (Fig. 6). Sample k_g_04 from bioproject PRJNA644779 contained an ARG against lincosamides while the gene found in sample y_g_01 is responsible for resistance against rifamycin. Contigs harbouring these ARGs had the best alignment to *L. lactis* and *B. animalis*, respectively. Bioproject PRJNA388572 had two samples with similar matches, except for gene *mdtB* which appeared in full length in sample k_p_14 and was absent in k_p_15, this gene is responsible for aminocoumarin resistance. As some other ARGs of the sample also have the potential to confer resistance against aminocoumarin, the AMR profiles of the samples appeared to be the same, including aminocoumarin, aminoglycoside, carbapenem, cephalosporin, cephamycin, diaminopyrimidine, elfamycin, fluoroquinolone, fosfomycin, glycylcycline, macrolide, monobactam, nitrofuran, nitroimidazole, penam, penem, peptide, phenicol, rifamycin, tetracycline and triclosan resistance. Comparing this list to the nudged results oxazolidinone, pleuromutilin and streptogramin resistance genes were absent. Contigs containing ARGs had the best alignment to the genome of *E. hormaechei* in both cases^8^.

All four samples of both bioprojects included at least 20 million bacterial reads in the assembly of the contigs. The other samples consisted of significantly fewer reads. Consequently, as Sims et al.^9^ found, it is not possible to distinguish whether the absence of protein-coding genes or the disruption of open reading frames (ORFs) represent a deficiency of the assembly or real evolutionary gene loss. Examining the relationship between the number of bacterial reads and the length of identified ARGs (including nudges) with a linear model we found that after each extra 100,000 reads the coverage of reference gene raises by 7% by the ARG coding ORFs ($p<0.0001$). In samples k_g_04, k_p_14, k_p_15 és a y_g_01, we randomly chose the average read number of the other samples (677,340) to reanalyze how much these results differ from the original ones executed on the full database. Contigs assembled contained one gene that was identified previously (excluding nudges), namely *lmrD* from sample k_g_04. ORFs predicted to be ARGs had a median coverage of 16.10% on the reference ARG sequences. In contrast, ORFs aligning to ARGs composed of the full read content of the four samples had a median coverage of 99.21%.

## Discussion

Studying ARGs that may enter the body with food, including fermented dairy products, can lead to critical health considerations. Characteristics of bacterial diversity and ARG abundance are well observable both in kefir and in yoghurt. ARG abundance is much higher in kefir than in yoghurt. One possible reason for this phenomenon could be the presence of fungi in kefir seed cultures. Since fungi may produce antibacterial toxins, bacteria having ARGs may gain a competitive advantage when coexisting with fungi.

Each bacterium (*Bifidobacterium animalis*^10^, *Enterobacter hormaechei*^8,11^, *Lactobacillus acidophilus*^11,12^, *Lactobacillus delbrueckii*^11,12^, *Lactobacillus helveticus*^11,12^, *Lactobacillus kefiranofaciens*^11–13^, *Lactobacillus plantarum*^11,12,14^, *Lactococcus lactis*^11,12,15,16^, *Leuconostoc mesenteroides*^11,12,15,16^) obtained from the taxon classification of contigs containing ARGs is widely used in the production of fermented dairy products. Li et al.^17^ analyzed the ARG content of isolated bacteria from fermented drinks and yoghurts. According to their results, *APH(3”)-III*, *APH(6’)-APH(2”)*, *sul1*, *tet(M)* were detectable in *Lactobacillus bulgaricus* strains, while *APH(3”)-II*, *sul1*, *sul2*, *strA*, *strB*, *tet(M)* derived from *Streptococcus thermophilus*. In our study *APH(3’)-IIb* gene belonging to the APH gene family, supposedly originated from *L. mesenteroides*. Similarly, Carr et al.^18^ found a strong co-occurrence between *APH(3’)-Ia* and *L. mesenteroides* in Chinese saliva samples. Further similarity with the results of Carr et al.^18^ is that *lmrD* originated from *Lc. lactis*. Guo et al.^19^ detected ARGs in *Lactobacillus* strains of fermented milk products. They detected gene *erm(B)*, *gyrA*, *rpoB*, *vanE*, *vanX* in *Lactobacillus casei*, gene *APH(3”)-III*, *dfrD*, *erm(B)*, *gyrA*, *tet(W)*, *vanX* in *L. helveticus*, gene *erm(B)* and *vanX* in *L. plantarum*. We found the *poxtA* gene associated with *L. helveticus* and *L. plantarum*. The *emrB* gene was identified in a contig from the genome of *E. hormaechei*.

During the fermentation of milk, the bacteria in seed cultures (and in milk) multiply and dominate the beverage. If any of these bacteria harbour ARGs, the amount of these genes will be increased in the final products. Based on data generated by Walsh et al.^7^ *L. helveticus* and *L. kefiranofaciens* are the most probable origin of the contigs harbouring gene *poxtA*. Sequences containing gene *APH(3’)-IIb* could have been stemmed from *L. mesenteroides*. According to Walsh et al.^7^, during fermentation the relative abundance of *L. kefiranofaciens* and *L. mesenteroides* increased. Not surprisingly, in our reanalysis of the same data we found the same trends (Fig. 5b). While Marsh et al.^20^ presented same changes of these species in kefir, Wurihan et al.^21^ showed opponent alterations in koumiss fermentation. ARG abundances (Fig. 5a) showed a positive association with the relative abundances of their most probable bacteria of origin (Fig. 5b). An increase in the relative abundance of *L. mesenteroides* was followed by the *APH(3’)-IIb* abundance. In contrast, *poxtA* abundance dropped simultaneously with the decrease of the relative abundance of *L. kefiranofaciens*.

The two most abundant ARGs were *poxtA* and *APH(3’)-IIb*, which were both presents in yoghurt and kefir samples. *PoxtA* (phenicol-oxazolidine-tetracycline resistance gene), an abundant ARG in Gram-positive bacteria, confers resistance to a wide range of critical antibiotics. The ABC-F class ATP binding ribosomal protection protein encoded by this gene is mainly present in Enterococci and Staphylococci. It was also identified in a methicillin-resistant *Staphylococcus aureus* (MRSA) strain that showed increased MIC to linezolid, a member of the oxazolidine class of antibiotics^22^. The study highlighted that Staphylococci, Enterococci and interestingly, *Pediococcus acidilactici* harbouring the gene are all of animal origin and can be spread horizontally with the help of mobile genetic elements. In line with other paper^23^ the study suggests that phenicols and other antiribosomal agents used in veterinary medicine might have played a role in the selection of *poxtA*. This was also confirmed by Elghaieb et al.^24^, who identified the gene in cow milk and animal wastewater. As oxazolidines are prohibited in food animals, and phenicols are not permitted in dairy cattle in Europe, the source of these genes in Hungarian samples remains to be elucidated. *Pseudomonas aeruginosa* harbours an array of aminoglycoside-modifying genes, altering the drug by acetylation, adenylation or phosphorylation (APH). The presence of *APH(3’)-IIb* in kefir samples is deliberately worrying as aminoglycoside 3’-phosphotransferases can mediate high-level resistance against several aminoglycosides. These genes might be plasmid-borne or chromosomally encoded; *APH(3’)-IIb* is the latter, but a transposon-mediated mechanism has been suggested to be responsible for spreading the resistance genes^25,26^. As the gene was almost exclusively described in *P. aeruginosa* previously, and the likely origin was *L. mesenteroides* in our study, the routes of resistance gene transfer related to this gene need to be further investigated. Although penicillins and cephalosporins are the most frequently used antibiotics for dairy cows, interestingly, the abundance of ARGs facilitating resistance against $\beta$-lactams is rather lacking. This phenomenon, together with the ARGs related to unused antibiotics in veterinary dairy medicine, raises the suspicion that the source of the abundant ARGs might not be a direct consequence of antibiotic use at dairy farms.

Bacteria entering the digestive tract with food, are provided with the opportunity of contacting other non-pathogenic and pathogenic bacteria. At the same time, one of the main prerequisites of HGT processes is the physical proximity of the participating bacteria. By virtue of the fulfilment of this requirement, various genes, including ARGs, can be exchanged by bacteria during horizontal gene transfer processes. If an ARG harbours on a mobile DNA-sequence, the probability of its HGT is higher. We found only one gene, namely *lmrD* in sample k_g_04, that is supposedly mobile. This deduction is based on the genomic environment of *lmrD*.

Antibiotic resistance caused by multidrug-resistant bacteria is a significant global public health threat^27^. Infections with drug-resistant bacteria may result in major morbidity and mortality and increase the cost of health care when compared to infections by non-resistant strains of the same species. Even with the strictest filtering restrictions, we identified ARGs undermining the efficacy of aminoglycosides, carbapenems, cephalosporins, cephamycins, diaminopyrimidines, elfamycins, fluoroquinolones, fosfomycins, glycylcyclines, lincosamides, macrolides, monobactams, nitrofurans, nitroimidazoles, phenicols, rifamycins and triclosans. These findings raise several clinical considerations. For instance, carbapenems are broad-spectrum antibiotics used for the treatment of necrotizing pancreatitis^28^ and severe intraabdominal infections. Tigecycline, a recently developed third-generation tetracycline antibiotic belonging to the glycylcycline class, is one of the few therapeutic options for carbapenem-resistant bacteria, like Klebsiella pneumoniae^29,30^ and carbapenem-resistant Enterobacteriaceae (CRE)^31^. Another group of ARGs identified in our study code resistance against fluoroquinolones. Emerging fluoroquinolone resistance in Campylobacter strains which are the leading cause of bacterial gastroenteritis in the world is a significant public health concern similarly to the rising incidence of fluoroquinolone-resistant cases of typhoid fever and invasive non-typhoidal Salmonella (iNTS) infections. We have also identified genes coding cephalosporin resistance in our samples. Cephalosporins belong to the most frequently used antibiotics globally. Intravenous third generation cephalosporins (e.g. ceftriaxone) are more potent against Gram-negative bacteria. They are frequently used in cholecystitis, spontaneous bacterial peritonitis or as a preventive measure in acute gastrointestinal haemorrhage^32^. ORFs harbouring ARGs that code resistance against macrolides may also raise serious public health concerns. Macrolide antibiotics absorb excellently from the gastrointestinal tract and have few side effects. Clarithromycin is still considered as a member of the first-line treatment protocol for Helicobacter pylori eradication in areas with a low resistance to clarithromycin^33^. Azithromycin can contribute to the resolution of acute infections by immunomodulatory effects^34^. It is frequently used for the treatment of acute watery or febrile diarrhoea and dysentery syndrome^35^. Tetracycline resistance genes that we found predestine a potential loss in the efficacy of various tetracycline compounds. Once commonly used, nowadays rarely administered tetracycline has been recently rediscovered, as a component of H. pylori eradication regimen, partly due to increasing rate of resistance to other antibiotics (including the above-mentioned clarithromycin)^33^.

As ARGs reaching the human body may originate from fermented dairy products, further examinations would be worthwhile to clarify the details and understand the practical medical significance. For this, it would be appropriate to analyze the samples of starter cultures and final products and register the results at set time points during the fermentation period. According to our findings, sequencing depth plays a significant role in the coverage of ORFs identified as ARGs, thus involving at least 20 million clusters is recommended by similar studies. The samples we examined and the studies we found in the literature^3–5,17,19^ confirm the hypothesis that foods of animal origin may contain significant amounts of diverse ARGs. The reason for the appearance of ARGs is complex, and the routes of appearance and spread are difficult to track. As sequencing techniques become cheaper, regular genetic monitoring of products of animal origin, including starter cultures, should be considered in addition to the strict control of antibiotics used in animal husbandry.

## Methods

### Data

The details of analyzed samples are listed in Table 1. One kefir and one yoghurt starter culture were shotgun sequenced (PRJNA644779) by the authors. The further short read datasets were obtained from NCBI SRA repository. A query was performed in SRA to find kefir or yoghurt related shotgun sequenced samples. As a result of this search further 33 datasets originating from 8 BioProjects were selected for the study. Except for the samples of BioProjects PRJEB15432 all others came from paired-end runs. The downloaded short reads originated from BioSamples of kefir grains (n = 4), kefir products (n = 15), kefir strains (n = 7), a yoghurt grain (n = 1) and yoghurt products (n = 5). In the unified names of the samples the first character corresponds to the type of the sample (k and y, kefir and yoghurt, respectively), the second character comes from the first letter of the source (g, p and s for grain, product and strain, respectively), while the last tag is a sequence number. Of the collected projects, a peer-reviewed publication is available for the PRJNA222257^36^, PRJEB15432^7^ and PRJEB30083^37^. For all other samples, the only accessible metadata were the attributes in SRA. In PRJEB15432 Walsh et al.^7^ followed the microbial changes during the fermentation process of kefir. They used full-fat pasteurized milk inoculated by three different grains (Fr1, Ick, and UK3 from France, Ireland and United Kingdom, respectively). The pasteurized milk (with three replications) and grains (without replication) were sampled at hour 0. In the fermentation from kefir at hour 8 (without replication) and hour 24 (with three replications), further specimens were taken.

**Table 1:** The list of analyzed samples obtained from NCBI SRA.

Sample ID	BioProject	Run	Type	Source	Sample
k_g_01	PRJEB15432	ERR1653138	kefir	Grain	Fr1 grain
k_g_02	PRJEB15432	ERR1653139	kefir	Grain	Ick grain
k_g_03	PRJEB15432	ERR1653140	kefir	Grain	UK3 grain
k_g_04	PRJNA644779	SRR12171332	kefir	Grain	kefir seed culture
k_p_01	PRJEB15432	ERR1653129	kefir	Product	UK3, 8 h
k_p_02	PRJEB15432	ERR1653130	kefir	Product	Fr1, 24 h (replicate 2)
k_p_03	PRJEB15432	ERR1653131	kefir	Product	Ick, 24 h (replicate 2)
k_p_04	PRJEB15432	ERR1653132	kefir	Product	UK3, 24 h (replicate 2)
k_p_05	PRJEB15432	ERR1653135	kefir	Product	Fr1, 24 h (replicate 3)
k_p_06	PRJEB15432	ERR1653136	kefir	Product	Ick, 24 h (replicate 3)
k_p_07	PRJEB15432	ERR1653137	kefir	Product	UK3, 24 h (replicate 3)
k_p_08	PRJEB15432	ERR1653141	kefir	Product	Fr1, 24 h (replicate 1)
k_p_09	PRJEB15432	ERR1653142	kefir	Product	Ick, 24 h (replicate 1)
k_p_10	PRJEB15432	ERR1653143	kefir	Product	UK3, 24 h (replicate 1)
k_p_11	PRJEB15432	ERR1653145	kefir	Product	Fr1, 8 h
k_p_12	PRJEB15432	ERR1653146	kefir	Product	Ick, 8 h
k_p_13	PRJNA288044	SRR2082409	kefir	Product	KEFIR.shotgun
k_p_14	PRJNA388572	SRR7287342	kefir	Product	Metagenome from probiotic beverage K03
k_p_15	PRJNA388572	SRR8282406	kefir	Product	Metagenome from probiotic beverage K02
k_s_01	PRJDB4955	DRR064132	kefir	Strain	*Lactobacillus parakefiri JCM 8573*
k_s_02	PRJNA222257	SRR1151211	kefir	Strain	*Lactobacillus kefiranofaciens subsp. kefiranofaciens DSM 5016*
k_s_03	PRJNA222257	SRR1151212	kefir	Strain	*Lactobacillus kefiranofaciens subsp. kefirgranum DSM 10550*
k_s_04	PRJNA222257	SRR1151213	kefir	Strain	*Lactobacillus kefiri DSM 20587*
k_s_05	PRJNA222257	SRR1151226	kefir	Strain	*Lactobacillus parakefiri DSM 10551*
k_s_06	PRJNA635855	SRR11965732	kefir	Strain	*Acetobacter syzygii str. K03D05*
k_s_07	PRJNA635872	SRR11966381	kefir	Strain	*Lactobacillus plantarum K03D08*
m_01	PRJEB15432	ERR1653133	milk	Milk	0 h (replicate 1)
m_02	PRJEB15432	ERR1653134	milk	Milk	0 h (replicate 2)
m_03	PRJEB15432	ERR1653144	milk	Milk	0 h (replicate 3)
y_g_01	PRJNA644779	SRR12171305	yoghurt	Grain	yoghurt seed culture
y_p_01	PRJEB30083	ERR2982980	yoghurt	Product	Yoghurt-A
y_p_02	PRJEB30083	ERR2982981	yoghurt	Product	Yoghurt-B
y_p_03	PRJEB30083	ERR2982982	yoghurt	Product	Yoghurt-C
y_p_04	PRJEB30083	ERR2982983	yoghurt	Product	Yoghurt-D
y_p_05	PRJEB30083	ERR2982984	yoghurt	Product	Yoghurt-E

In the unified names of the samples the first character corresponds to the type of the sample (k and y, kefir and yoghurt, respectively), the second character comes from the first letter of the source (g, p and s for grain, product and strain, respectively), while the last tag is a sequence number. The last column shows the available attribute data of the biosamples.

### DNA extraction and metagenomics library preparation for PRJNA644779

Total metagenome DNA of kefir (k_g_04) and yoghurt (y_g_01) samples were extracted using the UltraClean Microbial DNA Isolation kit from MoBio Laboratories. The quality of the isolated total metagenomic DNA was checked using an Agilent Tapestation 2200 instrument. The DNA samples were used for in vitro fragment library preparation. In vitro fragment libraries were prepared using the NEBNext Ultra II DNA Library Prep Kit for Illumina. Paired-end fragment reads were generated on an Illumina NextSeq sequencer using TG NextSeq 500/550 High Output Kit v2 (300 cycles). Read numbers were the following: 22,044,496 and 20,895,112 for kefir and yoghurt, respectively. Primary data analysis (base-calling) was carried out with Bbcl2fastq software (v2.17.1.14, Illumina).

### Bioinformatic and statistical analysis

Quality based filtering and trimming was performed by Trimmomatic^38^, using 15 as a quality threshold. Only reads longer than 50 bp were retained. The remaining reads were taxonomically classified using Kraken2 ($k=35$)^39^ with the NCBI non-redundant nucleotide database^40^ with two different confidence setting. The first run was performed with the default settings to select all possible bacterial reads. The following taxon classification was performed with the –confidence 0.5 parameter to get more precise species level hits. The taxon classification data was managed in R^41^ using functions of the package phyloseq^42^ and microbiome^43^. For further analysis, only reads assigned to Bacteria Kingdom was used^44^. The preprocessed bacterial reads were assembled to contigs by MEGAHIT (v1.2.9)^45^ using default settings. From the contigs, all possible open reading frames (ORFs) were gathered by Prodigal^46^. The protein translated ORFs were aligned to the ARGs of database CARD v.3.0.9^47,48^ by Resistance Gene Identifier (RGI, v5.1.0) with Diamond^49^. The ORFs classified as perfect or strict were further filtered with 90% identity and 60% coverage. The findings were presented including and excluding the nudged hits. For the analysis of ARG abundance changes during kefir fermentation, only ARGs with maximal coverage greater than 75% in samples taken at different time points were included. Contigs harbouring ARGs were classified by Kraken2 using the NCBI RefSeq^50^ complete bacterial genomes database. In keeping with Hendriksen at al.^1^ the ARG abundance was expressed as fragments per kilobase per million fragments (FPKM)^51^ of contigs containing ARGs. For the *i*th contig $FPKM_i=q_i/(l_i\times Q)\times 10^6$, where $q_i$ is the number of reads that mapped to the contig, $l_i$ is the length of contig and *Q* is the total number of mapped reads. To calculate *q* values, all bacterial reads were aligned to the contigs by Bowtie2^52^ with the parameter of –very-sensitive-local. To identify possible further mobile genetic element (MGE) homologs the predicted protein sequences of contigs were scanned by HMMER^53^ against data of PFAM v32^54^ and TnpPred^55^. Similar to Saenz et al.^44^ from the hits with lower than E $10^{-5}$ the best ones were assigned to each predicted protein within the distance of 10 ORFs. The MGE domains coexisting with ARGs were categorized as phage integrase, resolvase, transposase or transposon. The plasmid origin probability of the contigs was estimated by PlasFlow v.1.1^6^. According to the ARG abundance of the samples, a dissimilarity matrix was calculated using the Bray-Curtis index^56^ with package vegan^57^. With the same library and the same matrix, a permutational multivariate analysis of variance was applied to quantify the associations between the dissimilarity and independent variables (type, source, BioProject). For the visualization of the sample distances based on this matrix, a principal coordinate analysis (PCoA) was performed with package ape^58^. The relationship between the detected ORF length and the sequencing depth was explored using a linear model. All analyses and plotting were done in R-environment^41^.

## Supplementary information

Supplementary Tables

## Data Availability

The short read data of sample data are publicly available and can be accessed through the PRJDB4955, PRJEB15432, PRJEB30083, PRJNA222257, PRJNA288044, PRJNA388572, PRJNA635855, PRJNA635872, PRJNA644779 from the NCBI Sequence Read Archive (SRA).
